# Environmental Impact of Surgical Masks Consumption in Italy Due to COVID-19 Pandemic

**DOI:** 10.3390/ma15062046

**Published:** 2022-03-10

**Authors:** Antonella Cornelio, Alessandra Zanoletti, Stefania Federici, Luca Ciacci, Laura Eleonora Depero, Elza Bontempi

**Affiliations:** 1 INSTM and Chemistry for Technologies Laboratory, University of Brescia, via Branze 38, 25123 Brescia, Italy; a.cornelio001@unibs.it (A.C.); stefania.federici@unibs.it (S.F.); laura.depero@unibs.it (L.E.D.); elza.bontempi@unibs.it (E.B.); 2 Department of Industrial Chemistry “Toso Montanari”, Alma Mater Studiorum-University of Bologna, 40136 Bologna, Italy; luca.ciacci5@unibo.it; 3 Interdepartmental Centre for Industrial Research “Renewable Resources, Environment, Sea and Energy”, Alma Mater Studiorum-University of Bologna, 40136 Bologna, Italy

**Keywords:** COVID-19, face masks, environmental pollution, waste, CO_2_ emission, Carbon Footprint, SDGs

## Abstract

The COVID-19 pandemic suddenly changed the lifestyle of billions of people. Face masks became indispensable to protect from the contagion providing a significant environmental impact. The aim of this work is to propose possible solutions to decrease masks’ impact on the environment. For this reason, different masks (surgical and fabric) were considered, and the CO_2_ emissions associated with the mask materials production were calculated. Carbon Footprint (CF) for each material composing the masks was evaluated through the database Ces Selector 2019. The software Qgis (version 2.18.20) allows us to elaborate the CO_2_ emissions maps for each Italian region. Finally, for surgical masks, which are often imported from abroad, the CF related to transport was considered. It results that fabric masks are a sustainable solution to prevent contagion. The total CO_2_ emission associated with the use of fabric masks from the beginning of the pandemic (March 2020) to December 2021 resulted in about 7 kton compared to 350 kton for surgical masks.

## 1. Introduction

The outbreak of COVID-19 made people suddenly change their habits. The pandemic’s rapid spread forced governments to undertake restrictive measures to contrast the contagions [1]. The unexpected shutdown had different economic, social, and environmental consequences. Social distancing and the mandatory use of personal protective equipment (PPE) such as masks and gloves imposed by the pandemic to prevent contagion revolutionized the way of life and interactions between people [2]. Raw material supply chains suffered serious shortages [3]. As a consequence of industrial activities interruptions and people confinement, a significant decrease in the release of greenhouse gases (GHG) emissions occurred [4]. It was estimated that the global reduction in CO_2_ emission, comparing 2019 and 2020, was about 5%, corresponding to 2 billion tons CO_2eq_ [5]. Lockdown in Italy had mainly concerned industries, commercial and transportation activities, strictly connected with the consumption of energy and their associated GHG emissions [6]. Focusing the attention on March 2020, it is evident that all sectors suffered a decrease in CO_2_ emission except for domestic heating due to low temperature and people’s confinement [7].

On the other side, the production of PPE grew dramatically to meet the global demand. The World Health Organization (WHO) estimated that in 2020 the global production of masks and gloves was 129 and 69 billion per month, respectively [8]. Although the use of fabric masks has been allowed under certain circumstances [9], most face masks are produced from plastic polymers such as polypropylene (PP), polyethylene (PE) and polyurethane (PU), therefore relying on fossil sources for production. The proper disposal of face masks is extremely important as such a huge amount of disposable products may have severe consequences on the environment and human health [10]. Inadequate waste management contributes to COVID-19 diffusion [11].

In addition, it is clear that the use of masks for the COVID-19 emergency had not reduced plastic pollution [12]. Moreover, not only the use of PPE but also disposable products such as plastic products (like cutlery and plates) and packaging was encouraged to limit the spread of virus contagion [13]. This highlights the essential role of plastic in daily life. For instance, the use of plastic packaging increased by 31% for Italy and 78% for the US [14]. During the COVID-19 pandemic, there was an increase in disposable plastic [15]. This is even more evident considering that in 2018, the amount of plastics produced every year was estimated at about 360 million tons, of which 8 million tons reach the oceans, through incorrect disposal, wind, soil leaching or contaminated effluent [16]. Instead, in 2020 the amount of plastics increased dramatically to 698 million tons, particularly for gloves, masks, gowns and goggles [17]. According to United Nations Environment Program [18], about 75% of used masks and other pandemic-related waste end up in landfills or seas. It is estimated that a high quantity of plastic debris (from 0.15 to 0.39 million tons) could reach the ocean from the coastal region in one year [19]. Surgical masks are made of plastic polymers with very low biodegradability. Indeed, they take hundreds of years to degrade and during this process they fragment in microplastics due to different factors such as temperature, UV radiation, and mechanical processes, as reported in recent studies [17,20,21]. In particular, Saliu et al. [22] reveal that a surgical mask can release in the marine environment thousands of microscopic fibers that are potentially dangerous both for marine flora and fauna and for humans. On the contrary, fabric masks are generally made from cotton, which is more biodegradable and can be reused several times.

**Figure 1:**
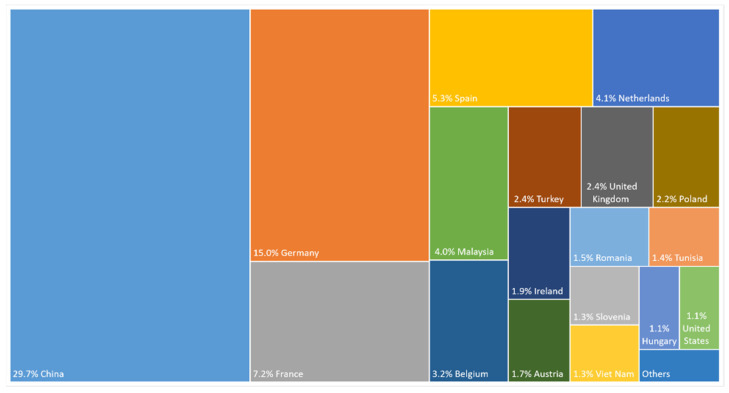
Euro values of imported personal protective equipment (PPE) in Italy, in 2020, expressed in %. Others includes the countries with % lower than 1%.

Further, indirect impacts associated with the COVID-19 pandemic concerned trade in PPE, which may become particularly significant at the national scale. For instance, a few days after the Italian government declared the lockdown on 9th March [23], PPE became unavailable and sold at very high prices because of the sudden and unexpected demand. To cope with this, many Italian factories converted their production to produce PPE, especially masks [24]. However, given the very high demand, which reached 1 billion masks and 0.5 billion gloves per month [25], Italy had to rely on imports of PPE from different countries, mainly China (about 30% of total supply cost) and Germany (15%) as shown in Figure 1 [26].

Although the supply of PPE is an essential means to constrain COVID-19 outbreaks, the environmental burden of this material requirement has been little investigated. The aim of this work is to propose possible solutions to decrease masks’ impact on the environment. To this purpose we assessed the potential contribution to global warming resulting from face masks consumption in Italy from March 2020, when masks became mandatory, until December 2021, focusing the attention on surgical and fabric masks. Surgical masks were the first to be used and certainly the most used thanks to their lower price. Fabric masks are the easiest to produce, even homemade, with very low costs. Quantitative estimation of GHG emissions includes direct and indirect CO_2eq_ release from material production and preliminary estimates for import for the surgical masks from trade partners.

## 2. Materials and Methods

**Scheme 1:**
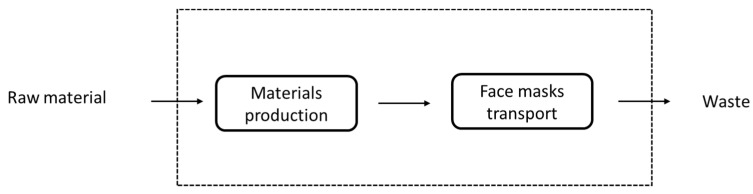
System boundary considered in this work.

For the evaluation of the Carbon Footprint (CF) due to face masks consumption, in this work, we have considered the Eco-audit approach, proposed by Ashby [27]. It considers the main lifecycle steps of a product: materials production, product manufacturing, transport, product use, and potential end-of-life credits. Due to the limited operations of materials assembly, the product manufacturing contributing to CF values can be neglected. The transport contribution can be evaluated by considering the product origin. Finally, no end-of-life credits can be considered because it is not considered to recycle face masks at the end of their life. The Ces Selector 2019 (Granta Design, Cambridge, UK) [28] was used to quantify the environmental impact of face masks in terms of CF. The Ces database contains all the materials of CF contribution, i.e., CF due to the production of the materials. The Eco-audit tool of the Ces Selector is a simplified approach, compared to life cycle assessment (LCA). It requires less data input to obtain a preliminary evaluation of the environmental impact of materials and processes [29]. On the contrary, LCA is a complex and onerous procedure that requires the knowledge of extensive data to evaluate the full process from extraction of raw material to disposal [30]. Scheme 1 represents the system boundary considered in this work.

To calculate the CO_2_ emissions (*E*) in Italy, expressed in tonCO_2_, related to the materials composing masks, the following data were considered:

- the weight of the mask, distinguishing from the material they were made of;
- the CO_2_ emissions of each material of which the masks were composed, expressed in kgCO_2_/kg of material. These values were provided by the software Ces Selector;
- calculation of the emission for a single mask (*e*), expressed in tonCO_2_;
- number of people using it assuming different scenarios (presented in Section 2.1 and Section 2.2).

**Figure 2:**
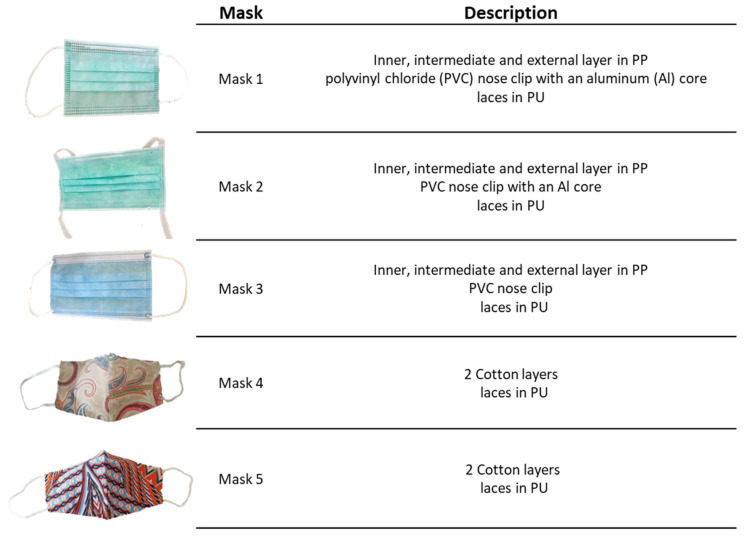
Different masks typologies: three different surgical masks, two different fabric masks.

Figure 2 shows face masks considered in this work and their characteristics: three different surgical masks, called mask 1, mask 2 and mask 3 compared with two fabric masks, mask 4 and mask 5.

To be safe for human health, surgical masks must be produced in compliance with the requirements of the standard UNI EN 14683:2019 defining the materials to be used and the requirements in terms of breathability, bacterial filtration efficiency and resistance to liquid splashing [31]. All three surgical masks are characterized by three layers, with dimension 15 × 15 cm^2^ (without pleating), in PP: the external layer, manufactured with spunbond technology, is water-resistant; the intermediate layer, produced with meltblown technology (at least 20 g/m^2^), is the filtering layer; and the inner layer, produced with spunbond technology, has a protective function avoiding the direct contact of the skin with the intermediate filter layer.

Fabric masks are not subject to any legislation for approval; however, the Italian Institute of Health provides some tips for their correct production [32]. Mask 4 and mask 5, handmade by a local producer (Brescia, Italy), consist of two layers of cotton fabric, with dimensions 7 × 18 cm^2^ and 5.5 × 16 cm^2^, respectively. The difference between these two masks is that mask 4 has the laces (made of PU) wider than mask 5.

Data on the Italian population, divided by region, were found on the Istat (National Institute of Statistics) website, considering data on 1 January 2020 [33]. 

Qgis software (version 2.18.20) [34] was used to create Qgis maps for the evaluation of CO_2_ emissions per Italian region related to the use of face masks.

### 2.1. Italian Surgical Masks Impact

To quantify the number of people required to wear the mask, the Italian population was divided by age. According to legislation [35], children under 6 years are not required to wear masks, so they were not considered in this study. The rest of the population was divided according to working conditions, distinguishing between workers, older than 15 years, and non-workers. The latter category includes both unemployed and inactive persons (pensioners and persons, such as students, who do not seek employment). Data were taken for each Italian region. To calculate the number of masks needed for each person, some hypotheses were made. The mask should be changed every 8 h, so for workers it was considered two masks per day, for 5 days/week, and one mask/day during the weekend. For non-workers, four masks/week were considered. With all these obtained data it is possible to calculate the total emissions associated with the mask materials with Equation (1):

(1) $$E=P_{1}*w*n_{1}*e+P_{2}*w*n_{2}*e\;$$

Where, *E* is CO_2_ emission in tonCO_2_; *P*_1_ is workers (age ≥ 15 years); *P*_2_ is unemployed and inactive people; *w* is the weeks considered since the use of masks became mandatory (March 2020) until the end of December 2021, 95 weeks; *n*_1_ is the number of masks/week for workers; *n*_2_ is the number of masks/week for unemployed and inactive people; *e* is the emission for a single mask in tonCO_2_. The minimum and maximum values of CO_2_ emissions in tonCO_2_ for each region were reported in Table S1 of Supplementary Materials.

### 2.2. Italian Fabric Masks Impact

To quantify the CO_2_ emissions related to fabric masks, Istat data about the Italian population were considered without the distinction of the employment situation. The total emissions associated with the use of masks was evaluated with Equation (2):

(2) E=P∗w∗n∗e 

where, *E* is CO_2_ emission in tonCO_2_; *P* population not considering children under 6 years old; *w* is the weeks considered since the use of masks became mandatory (March 2020) until the end of December 2021, 95 weeks; *n* is the number of masks/week; *e* is the emission for a single mask in tonCO_2_. The minimum and maximum values of CO_2_ emissions in tonCO_2_ for each region were reported in Table S2 of Supplementary Materials. 

## 3. Results and Discussion

### 3.1. Surgical Masks

Masks were cut and each part weighed to obtain the weight of the individual components. The results, expressed in g, are reported in Table 1.

Knowing the different mask materials, the software Ces Selector provided CO_2_ emission data distinguishing between the minimum and maximum value, reported in Table 2.

**Figure 3:**
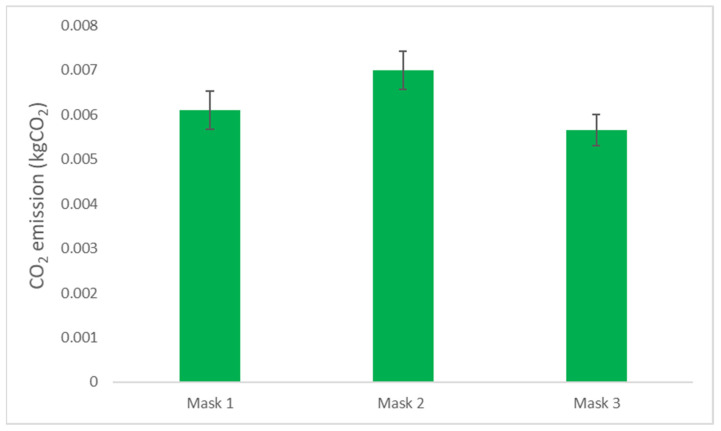
Total average CO_2_ emission for each surgical mask expressed in kgCO_2_. Error bars represent minimum and maximum values.

The CO_2_ emissions per mask (*e*) were calculated by multiplying the weight of the individual parts by the values obtained from the software Ces Selector. Data are reported in Figure 3, expressed in kgCO_2_.

### 3.2. Fabric Masks

Mask 4 and mask 5 were cut to weigh the single part. Data are reported in Table 3.

The Ces Selector provided CO_2_ emission data distinguishing between the minimum and maximum values, reported in Table 4.

**Figure 4:**
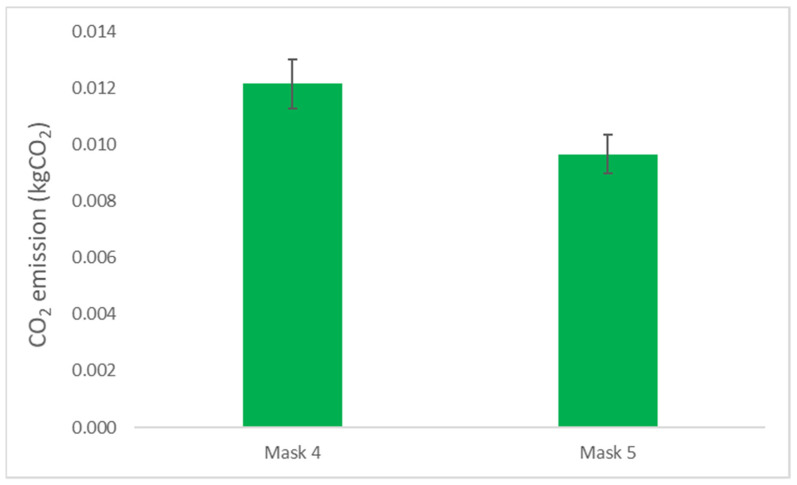
Total average CO_2_ emission for each fabric mask expressed in kgCO_2_. Error bars represent minimum and maximum values.

CO_2_ emissions per mask (*e*) were calculated as defined in Section 2. Results are reported in Figure 4.

The higher value of CO_2_ emission is attributed to the higher weight of fabric mask core compared to surgical mask. This apparent inconsistency is due to the fact that values reported in Figure 3 and Figure 4 are not normalized by the total weight of a single mask. However, according to the CO_2_ footprint, the materials used in the production of surgical masks have a higher impact compared to fabric masks (as reported in Table 2 and Table 4). In addition, plastic materials used for surgical masks are less biodegradable than cotton. Moreover, cotton masks have a longer life, can be washed and reused. According to the Italian National Institute of Health, these masks must be washed at 60 °C, the maximum number of washings is indicated by the manufacturer [35,36]. In this work, about 50 washes were considered before fabric mask replacement, so it can be expected that each individual uses about seven masks per year.

### 3.3. CO_2_ Emission in Italy Due to Face Masks Consumption

Between 2019 and 2020, the lockdown due to the pandemic allowed us to reach a decrease of about 10% in CO_2_ emissions in Italy [37]. The largest decrease, almost 18,000 kton, was found by comparing the emissions of March and April 2019 with those of the same period of the following year [6]. In particular, the region with the greatest decrease, about 3000 kton, was Lombardy [6].

From March 2020, face masks have become an object of daily use to prevent infection by COVID-19. Their impact on the environment cannot be neglected. In this study, we focused our attention mainly on CF due to the materials used for face masks realization. As reported in the literature, most CO_2_ emissions are associated with masks production [38]. For this purpose, CO_2_ emissions for all Italian regions were evaluated, as defined in Section 2.1 and Section 2.2.

**Figure 5:**
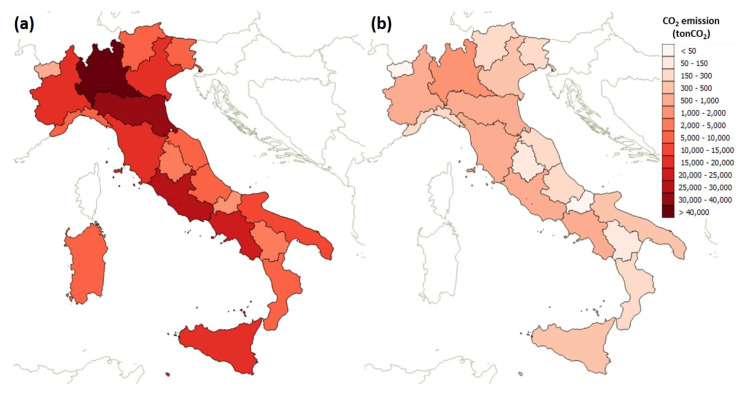
Qgis geographical visualization of CO_2_ emissions (*E*) associated with: (**a**) surgical masks consumption; (**b**) fabric masks consumption. Data were calculated considering the period from March 2020 to December 2021. These data were evaluated only considering the materials used. Carbon footprint associated with transport of surgical masks from abroad cannot be evaluated for each region (however a global value of 59 kton of CO_2_ per year due to the air transport was estimated [28]. Carbon Footprint associated with the transport of fabric masks is not considered in the analysis.

CF maps were elaborated through Qgis software and reported in Figure 5. Mask 2 and mask 4, which had the higher values of CO_2_ emissions, respectively, for surgical and fabric masks, were considered for the elaboration of maps. CO_2_ emission values for each region are reported in Tables S1 and S2 of Supplementary Materials.

It is evident that the higher number of surgical masks necessary to satisfy their correct use led to higher CO_2_ emissions related to all Italian populations. The total CO_2_ emissions associated with the use of surgical masks for all Italian regions from March 2020 to December 2021 corresponds to 240 kton. The CF associated with surgical mask materials is comparable to the CO_2_ emissions associated with solid fossil fuels burning during cold months (from November to March) [6]. The highest value is in Lombardy, followed by Lazio and Emilia-Romagna, as it is the most populated region in Italy and with a higher level of employment compared to the other regions. While the CO_2_ emissions value embodied in fabric masks is about 7 kton, almost 35 times less than the value related to surgical masks.

However, in Figure 5 only the CO_2_ emitted considering face masks materials were considered, neglecting the emissions due to other processes such as transport. Indeed, even if some Italian factories converted their production to produce surgical masks, most of them are still imported from abroad (mainly from China). For example, considering surgical masks imported from China and assuming that the transport takes place by air, it was already estimated that about 59 kton of CO_2_ are generated every year to import surgical masks [28]. Considering this data, it is possible to estimate a total CF associated with surgical masks use from March 2020 to December 2021 of about 350 kton of CO_2_. Local transport to bring the masks to the various shops was not considered.

On the other hand, fabric masks are easier to produce and are often packaged in local shops, in accordance with government directives [36]. All this surely has a smaller impact on the phase of transport reducing the emissions due to this phase.

It is evident that the COVID-19 epidemic can worsen the already serious environmental situation. All this has consequences on the achievement of the SDGs established by the 2030 Agenda [3], in particular the achievement of goals 6, 11, 12 and 14. Goals 6 and 14 concern clean water, sanitation and water below. The achievement of these goals is extremely important since, if not properly disposed of, face masks can cause serious damage to the aquatic ecosystem as they can release microplastics. As discussed in Section 1, surgical masks are made of PP, PE, PU and PVC, not so biodegradable. Then the massive use of surgical masks represents a significant environmental problem. It is clear that a solution to surgical masks could be fabric masks as cotton is a more biodegradable material. It is estimated that the degradation of cotton in water is more than 95% of the PE [39].

Goal 12 concerns responsible consumption and production. It is therefore of fundamental importance, to protect the planet from degradation, to make the population aware of responsible consumption and production patterns that promote the use of resources and renewable energy. The common objective is to aim for a longer life of consumer goods and to ensure that the latter are mostly made of recyclable material. Increased recycling of materials will help meet some of the circular economy goals, reducing dependence on scarce resources and mitigating permanent waste disposal to achieve sustainable cities (Goal 11) [3]. Moreover, it is necessary to take into account the large amount of waste produced by their use. Considering the weights of the three surgical masks in this work, the average weight is about 2.9 g. Following the calculations made to establish the number of surgical masks needed by the Italian population, every week are necessary 360 million masks. By multiplying this value with the average mask weight, we get that every week about 1000 tons of waste are produced increasing the volume of waste treated by incinerators. This waste is potentially hazardous. If the masks are used by persons positive to COVID-19, they cannot be treated as municipal waste but as hazardous waste comparable to those from health facilities. In this case, they should be disposed of in special incineration plants of which there are eight throughout Italy, according to Ispra [40]. In fact, if not properly disposed of they can be a contagion source as well [41]. It is evident that the high production and use of surgical masks have consequences on the environment, for the generation of a large amount of plastic waste, for specific treatments to which they must be subjected during the disposal phase and often for their incorrect disposal. In order to minimize risks related to their incorrect disposal, recent studies are proposing some masks disinfection treatments such as the use of hydrogen peroxide or UV light [14].

Concrete actions must be taken to minimize the environmental consequences of using masks. Fabric masks may represent a valid alternative as they are reusable and washable in the washing machine at 60 °C as reported by the Ministry of Health [36]. Emissions from washing masks have not been assessed as they could be washed with laundry. Nevertheless, important companies producing washing machines are moving in this direction by setting up specific washing cycles to disinfect them [42]. The use of fabric masks would reduce the problem of disposal and abandonment of PPE. Cotton is a natural fiber and is more easily biodegradable than the components of surgical masks which take hundreds of years to degrade.

## 4. Conclusions

The COVID-19 pandemic caused a global crisis with social, economic and environmental consequences. People suddenly changed their habits and face masks became a necessary accessory to protect from the contagion. Their massive use and often incorrect disposal caused a severe impact on the environment. In this study, the attention was focused on the CO_2_ emission due to their use. Two different typologies of face masks were compared: surgical and fabric masks. First of all, CF of single material composing masks was evaluated, then an estimate of the number of masks needed by the Italian population was made. Finally, CO_2_ emissions due to their use were evaluated for all Italian regions. It was estimated that the total CO_2_ emissions associated with the materials used for surgical masks from March 2020 to December 2021 are about 240 kton, compared to 7 kton of fabric masks. If the transport contribution is also considered, 350 kton of CO_2_ are estimated to be originated by surgical masks use. Although the approach used in this work is simplified compared to LCA analysis, it allows us to obtain a preliminary evaluation of the environmental impact. It is evident that surgical masks have a higher environmental impact due to the increased number of masks needed to meet the demand and use of poorly degradable materials. Moreover, surgical masks are specially imported from abroad, increasing CO_2_ emissions due to their use and transport. While, fabric masks are made of cotton, more degradable and reusable. Moreover, they are often produced locally reducing the transport component.

According to recent studies proposing different disinfection methods, for the future, it could be possible to evaluate the recovery of surgical masks to minimize the impact on the environment.

## Figures and Tables

**Table 1:** Weights of material components of masks. Values are reported in g.

Material	Mask 1	Mask 2	Mask 3
PP (g)	2.1	3	2.2
PU (g)	0.3	-	0.4
PVC (g)	0.1	0.1	0.3
Al (g)	0.1	0.1	-
Total (g)	2.6	3.2	2.9

**Table 2:** CO_2_ emissions, expressed in kgCO_2_/kg Material, for each material masks are made obtained by the software Ces Selector.

Material	CO_2_ Emission (kg CO_2_/kg Material)	
	Min	Max
PP	1.7	1.9
PU	3.1	3.4
PVC	2.1	2.3
Al	11	12

**Table 3:** Weights of masks in g.

Material	Mask 4	Mask 5
Cotton (g)	9.5	9.4
PU (g)	1.2	0.4
Total (g)	10.7	9.8

**Table 4:** CO_2_ emissions, expressed in kgCO_2_/kg Material, for each material masks are made obtained by the software Ces Selector.

Material	CO_2_ Emission [kg CO_2_/kg Material]	
	Min	Max
Cotton	0.9	1
PU	3.1	3.4

## Data Availability

Data is contained within the article.

## Supplementary Materials

The following supporting information can be downloaded at: https://www.mdpi.com/article/10.3390/ma15062046/s1, Table S1: Min and max values of CO_2_ emissions for each Italian region associated to surgical masks, expressed in tonCO_2_; and Table S2: Min and max values of CO_2_ emissions for each Italian region associated to fabric masks, expressed in tonCO_2_.
