# Supplementary material for: Environmental Impact of Surgical Masks Consumption in Italy Due to COVID-19 Pandemic
Source: Materials (Basel). 2022 Mar 10;15(6):2046. doi: 10.3390/ma15062046 (PMC8949017; doi:10.3390/ma15062046)
Supplement: Supplementary file 1 [file materials-15-02046-s001.zip › materials-1569515-supplementary.pdf]

materials

Environmental impact of surgical masks consumption in Italy due to COVID-19 pandemic

Antonella Cornelio[1], Alessandra Zanoletti[1]*, Stefania Federici[1], Luca Ciacci[2,3], Laura Eleonora Depero[1] and Elza Bontempi[1]

[1] INSTM and Chemistry for Technologies Laboratory, University of Brescia, via Branze 38, 25123 Brescia, Italy; a.cornelio001@unibs.it (A.C.); stefania.federici@unibs.it (S.F.); laura.depero@unibs.it (L.E.D.); elza.bontempi@unibs.it (E.B.)
[2] Department of Industrial Chemistry "Toso Montanari", Alma Mater Studiorum-University of Bologna, 40136 Bologna, Italy; luca.ciacci5@unibo.it
[3] Interdepartmental Centre for Industrial Research "Renewable Resources, Environment, Sea and Energy", Alma Mater Studiorum-University of Bologna, 40136 Bologna, Italy
* Correspondence: alessandra.zanoletti@unibs.it

Table S1. Min and Max values of CO_2 emissions for each Italian region associated to surgical masks, expressed in tonCO_2.

	**Surgical mask**					
	**Mask 1**		**Mask 2**		**Mask 3**	
**Region**	min CO_2 emission (tonCO_2)	max CO_2 emission (tonCO_2)	min CO_2 emission (tonCO_2)	max CO_2 emission (tonCO_2)	min CO_2 emission (tonCO_2)	max CO_2 emission (tonCO_2)
Piedmont	15,864	17,511	18,131	20,016	14,603	16,130
Aosta Valley	482	532	551	608	444	490
Lombardy	39,167	43,233	44,764	49,417	36,054	39,822
Trentino-South Tyrol	4367	4821	4991	5510	4020	4440
Veneto	16,330	18,025	18,663	20,603	15032	16,603
Friuli-Venezia Giulia	4524	4993	5170	5708	4164	4599
Liguria	5333	5887	6095	6729	4909	5423
Emilia-Romagna	17734	19,576	20,269	22,375	16325	18,031
Tuscany	14,057	15,517	16,066	17,736	12940	14,293
Umbria	3159	3487	3611	3986	2908	3212
Marche	5548	6124	6341	7000	5107	5641
Lazio	20,758	22,913	23,724	26,190	19108	21,105
Abruzzo	4341	4792	4962	5477	3996	4414
Molise	951	1050	1087	1200	876	967
Campania	14,325	15,812	16,372	18,073	13186	14,564
Apulia	10,857	11,984	12,408	13,698	9994	11,038
Basilicata	1659	1831	1896	2093	1527	1687
Calabria	4631	5112	5293	5843	4263	4709
Sicily	11,853	13,084	13,547	14,955	10911	12,052
Sardinia	5092	5620	5819	6424	4687	5177

Table S2. min and max values of CO_2 emissions for each Italian region associated to fabric masks, expressed in $tonCO_2$.

**Region**	**Fabric mask**			
	**Mask 4**		**Mask 5**	
	min CO_2 emission ($tonCO_2$)	max CO_2 emission ($tonCO_2$)	min CO_2 emission ($tonCO_2$)	max CO_2 emission ($tonCO_2$)
Piedmont	544	600	431	475
Aosta Valley	16	18	13	14
Lombardy	1339	1476	1061	1170
Trentino-South Tyrol	149	165	118	130
Veneto	471	519	373	411
Friuli-Venezia Giulia	154	170	122	135
Liguria	182	201	145	159
Emilia-Romagna	606	669	481	530
Tuscany	480	529	380	419
Umbria	108	119	86	94
Marche	190	209	150	166
Lazio	710	784	563	621
Abruzzo	148	164	118	130
Molise	33	36	26	28
Campania	490	541	389	429
Apulia	370	408	293	324
Basilicata	57	63	45	50
Calabria	160	177	127	140
Sicily	406	448	322	355
Sardinia	175	193	139	153
